# Structure of the Receptor Binding Domain of EnvP(b)1, an Endogenous Retroviral Envelope Protein Expressed in Human Tissues

**DOI:** 10.1128/mBio.02772-20

**Published:** 2020-11-17

**Authors:** Kevin R. McCarthy, Joseph L. Timpona, Simon Jenni, Louis-Marie Bloyet, Vesna Brusic, Welkin E. Johnson, Sean P. J. Whelan, Lindsey R. Robinson-McCarthy

**Affiliations:** a Department of Biological Chemistry and Molecular Pharmacology, Harvard Medical School, Boston, Massachusetts, USA; b Laboratory of Molecular Medicine, Boston Children's Hospital, Harvard Medical School, Boston, Massachusetts, USA; c Department of Microbiology, Harvard Medical School, Boston, Massachusetts, USA; d Department of Molecular, Cellular and Developmental Biology, University of Colorado, Boulder, Colorado, USA; e Department of Molecular Microbiology, Washington University School of Medicine, St. Louis, Missouri, USA; f Department of Biology, Boston College, Chestnut Hill, Massachusetts, USA; g Department of Genetics, Harvard Medical School, Boston, Massachusetts, USA; Virginia Polytechnic Institute and State University

**Keywords:** endogenous retrovirus, envelope protein, paleovirology, structure, evolution

## Abstract

EnvP(b)1 is an endogenous retroviral envelope gene found in human and other primate genomes. We report EnvP(b)1 sequences in primate genomes consistent with an integration event between 40 and 71 million years ago. Using a highly specific polyclonal antiserum raised against the putative receptor binding domain (RBD) of human EnvP(b)1, we detected expression in human placenta, ovaries, and thymus. We found that EnvP(b)1 is proteolytically processed, and using cell-cell fusion assays in multiple primate cell lines, we demonstrated that extant EnvP(b)1 proteins from a variety of primate genomes are fusogenic. This work supports the idea that EnvP(b)1 is under purifying selection and its fusogenic activity has been maintained for over 40 million years. We determined the structure of the RBD of human EnvP(b)1, which defines structural similarities with extant leukemia viruses, despite little sequence conservation. This structure highlights a common scaffold from which novel receptor binding specificities likely evolved. The evolutionary plasticity of this domain may underlie the diversity of related Envs in circulating viruses.

## Abstract (precis)

Organisms can access genetic and functional novelty by capturing viral elements within their genomes, where they can evolve to drive new cellular or organismal processes. We demonstrate that a retroviral envelope gene, EnvP(b)1, has been maintained and its fusion activity preserved for 40 to 71 million years. It is expressed as a protein in multiple healthy human tissues. We determined the structure of its inferred receptor binding domain and compared it with the same domain in modern viruses. We found a common conserved architecture that underlies the varied receptor binding activity of divergent Env genes. The modularity and versatility of this domain may underpin the evolutionary success of this clade of fusogens.

## INTRODUCTION

Integration into nuclear DNA is an obligate step in the retrovirus replicative cycle. Integration events in gamete cells or their progenitor cells can allow viral elements to be vertically transmitted to offspring. Approximately 8% of the human genome is derived from such endogenous retroviruses (ERVs), and similar percentages are observed across the animal kingdom (1). Most ERV loci have acquired inactivating mutations that erode the coding potential of individual genes and render these loci incapable of producing infectious viruses. However, some ERV genes can retain their function in an otherwise degraded provirus, an indication that selective pressures preserve the function of these genes. Over time, ERV genes can be coopted to perform beneficial or essential host functions. For example, the cell-cell fusion events that produce the placental syncytiotrophoblast layer in eutherian mammals are driven by multiple independently acquired and independently coopted ERV envelopes (Envs), known as syncytins (2). Primate genomes harbor a number of ERV Env open reading frames (ORFs) with unknown functions (3, 4). Many of these ORFs have been maintained for the duration of primate evolution, time periods exceeding 40 million years.

The human genome contains four copies of ERV-P(b), located on different chromosomes; one of these, located within an intron of the *RIN3* gene, retains an intact ORF termed EnvP(b)1 (5). The intact EnvP(b)1 ORF has been identified in Old World primates and must have arisen as the result of integration of a then-extant virus into the *RIN3* locus of a common primate ancestor. The primate EnvP(b)1 ORF is reportedly evolving under purifying selection, and various dating methods estimate EnvP(b)1 to be between 30 and 55 million years old (5, 6). EnvP(b)1 has the characteristics of a classical gammaretroviral envelope protein. All the sequence motifs necessary for processing and cell surface expression remain intact (5, 7). When ectopically expressed, human EnvP(b)1 can mediate fusion of cells in culture (7, 8). EnvP(b)1 RNA has been detected in human tissues; however, protein expression and processing have not been previously reported (5, 6). It is unclear what function(s) EnvP(b)1 may have that would explain the fact that it has maintained a functional ORF over tens of millions of years.

Here, we show that EnvP(b)1 protein is expressed in multiple human tissues in a form that appears to be fusion competent. We establish that EnvP(b)1 is more ancient than previously reported by identifying orthologs in the *RIN3* locus of apes, Old World monkeys and New World monkeys (simian species). Its absence in the syntenous locus of prosimian species indicates that EnvP(b)1 was acquired between 40 and 71 million years ago (MYA). The membrane fusogen activity of EnvP(b)1 has been preserved for the duration of simian evolution. The host factors required for fusion are conserved and are ubiquitously expressed in the cultured cells of primates. Thus, an activated, functional, ancient fusogen of unknown function is expressed in multiple healthy human tissues. We have determined the structure of the EnvP(b)1 inferred receptor binding domain and compared it with available structures of the homologous domain from extant retroviruses. Their similarities define a common structural architecture that has been retained, despite selection for different functions and divergence over timescales greatly exceeding 40 to 71 million years. Modularity within this domain demonstrates the flexibility of the architecture of this domain as a versatile scaffold for receptor binding.

## RESULTS

### EnvP(b)1 protein is expressed in human tissues.

**FIG 1:**
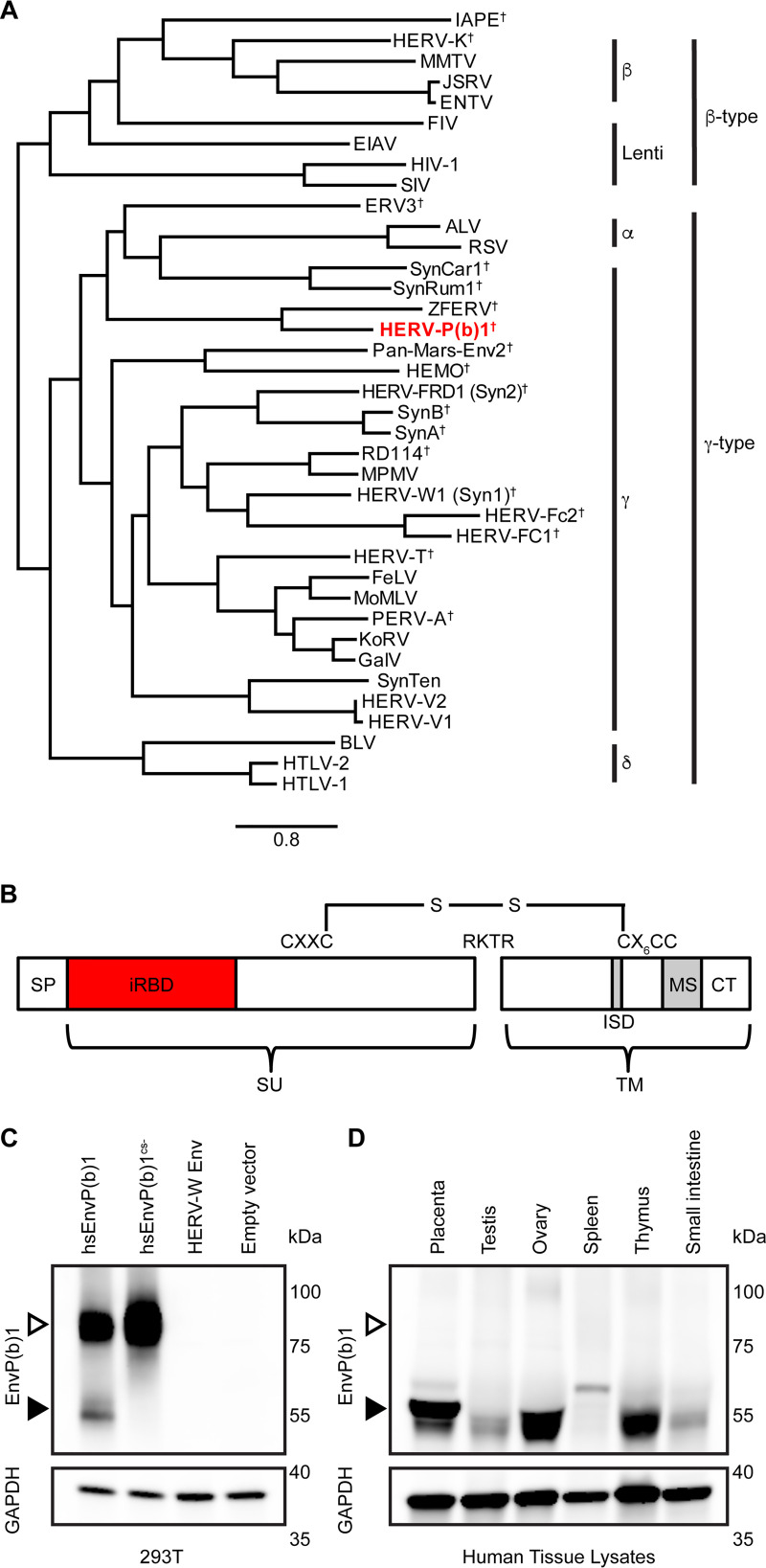
EnvP(b)1 protein is expressed and processed in human tissues. (A) Maximum-likelihood tree of retroviral Envs. Full-length amino acid sequences of Envs from five retroviral genera were used as inputs. The tree is rooted on IAPE (intracisternal A-type particle element with an envelope) Env. Genera of Env are indicated, as well as the type of Env based on phenotype and sequence (beta type and gamma type). Daggers indicate sequences that have been found only as endogenous sequences. EnvP(b)1 from human ERV-P(b) [HERV-P(b)1] is shown in red. The bar shows the scale, in amino acid substitutions per site. (B) Diagram of mature EnvP(b)1 protein. SU, surface subunit; TM, transmembrane subunit; SP, signal peptide; iRBD, inferred receptor binding domain; ISD, immunosuppressive domain; MS, membrane spanning domain; CT, cytoplasmic tail. The furin cleavage site (RKTR) is noted. (C) Lysates from 293T cells transfected with the indicated envelope were subjected to Western blotting with either anti-EnvP(b)1 serum or anti-GAPDH antibody. Open arrowheads indicate unprocessed SU-TM. Filled arrowheads indicate processed SU. (D) Expression of EnvP(b)1 protein in healthy human tissues. Protein lysates from the indicated tissues from healthy donors were subjected to Western blotting with either anti-EnvP(b)1 sera or anti-GAPDH antibody. A single band corresponding to EnvP(b)1 SU is present in all tissues except spleen.

EnvP(b)1 is the only remaining intact ORF in the ERV-P(b) provirus in the human *RIN3* locus, ERV-P(b)1. All other ERV genes at this locus have been disrupted. We produced a phylogeny to describe the evolutionary relationships of EnvP(b)1 with other retroviral Envs (Fig. 1A). EnvP(b)1 occupies a clade of viruses that comprises endogenous mammalian and extant avian examples. Based upon its phylogenetic relationships with other Envs and characteristic motifs in its surface (SU) and transmembrane (TM) domains, EnvP(b)1 has been classified as a gamma-type Env (5). Notable elements include an intact furin cleavage site and a CXXC/CX_6_CC disulfide bond motif (Fig. 1B).

EnvP(b)1 RNA has previously been detected in human tissues (5, 7). To determine whether EnvP(b)1 protein is also present, we expressed the genetically unique (no similar sequences in human or rabbit genomes) inferred receptor binding domain (iRBD) and used this iRBD protein to immunize a rabbit and produce antiserum. We enriched for EnvP(b)1 iRBD-binding IgGs with a series of affinity resins and confirmed by Western blotting that the enriched iRBD antisera detected EnvP(b)1 in transfected 293T cells (Fig. 1C). Two major bands were observed, one corresponding to the predicted molecular weight of unprocessed EnvP(b)1 SU-TM and a second corresponding to the furin-processed SU domain. The specificity of the SU band was confirmed by expressing EnvP(b)1 in which the furin cleavage site was mutated [EnvP(b)1^CS−^]. For this mutant, only the band corresponding to the unprocessed Env was observed. The reagent is specific for EnvP(b)1, as it failed to detect human syncytin-1 (HERV-W Env), and Western blots of mock-transfected 293T lysate did not show bands in the appropriate locations.

To determine whether EnvP(b)1 protein is expressed in healthy human tissues, we assayed tissue lysates by Western blotting under reducing conditions (Fig. 1D). We chose tissues that were similar to those in which RNA transcripts were previously detected (5, 7). Protein expression was strongest in placenta, ovary, and thymus. In these tissues, we detected a doublet band for SU that was most prominent in placenta. This may represent an additional proteolytic processing event or glycosylation heterogeneity. EnvP(b)1 protein was also detected at lower levels in testis and small intestine. A band of a different molecular weight was present in spleen lysate, which we therefore cannot conclude corresponds to EnvP(b)1 protein. Tissues from both healthy males and females showed clear EnvP(b)1 expression, indicating that expression is not restricted to females (Table S1). We did not observe a higher-molecular-weight species corresponding to unprocessed SU-TM in any tissue, suggesting that EnvP(b)1 is efficiently processed in these tissues. In contrast, overexpression of EnvP(b)1 in 293T cells produced primarily SU-TM (Fig. 1C).

10.1128/mBio.02772-20.7 TABLE S1 Information on the human tissue lysates probed for EnvP(b)1 expression. Tissue protein lysates were purchased from TaKaRa. Some lysates were from a single donor, and some were pooled from multiple donors. Information on the age, sex, and cause of death (COD) of the donors is presented. Donors were presumed to be healthy prior to death. Download Table S1, PDF file, 0.04 MB. Copyright © 2020 McCarthy et al. 2020 McCarthy et al. This content is distributed under the terms of the Creative Commons Attribution 4.0 International license.

Under nonreducing conditions SU remains covalently linked to TM via a disulfide bond, and we observed EnvP(b)1 as SU-TM and higher-molecular-weight species (Fig. S1A). In tissue lysates under nonreducing conditions, EnvP(b)1 was mainly detected as SU-TM and these higher-molecular-weight species (Fig. S1B). In these human tissues, SU-TM are covalently linked and EnvP(b)1 is likely to be in a state that is primed for fusion.

10.1128/mBio.02772-20.1 FIG S1 EnvP(b)1 is detected as higher-molecular-weight species under nonreducing conditions. (A) Western blot of cell lysates from A549 cells transfected with empty vector or human EnvP(b)1 under reducing (R) and nonreducing (NR) conditions. (B) Western blot of human tissues lysates under nonreducing conditions. Filled arrows indicate the position of SU, empty arrows indicate the position of SU-TM, and brackets indicate higher-molecular-weight species. Download FIG S1, TIF file, 1.0 MB. Copyright © 2020 McCarthy et al. 2020 McCarthy et al. This content is distributed under the terms of the Creative Commons Attribution 4.0 International license.

### EnvP(b)1 ORF is conserved in simian primates.

**FIG 2:**
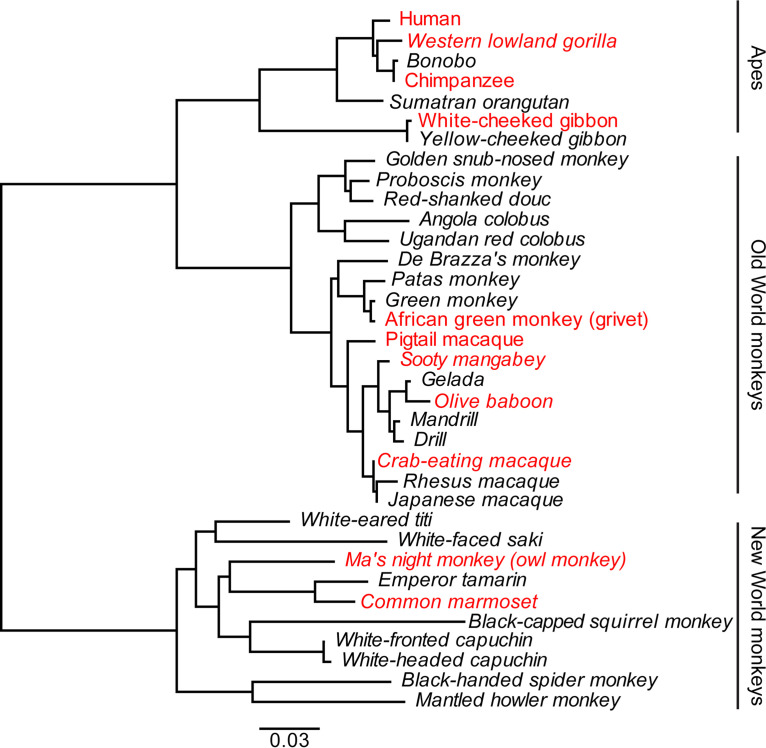
Phylogeny of EnvP(b)1 orthologs from the *RIN3* locus of simian primates. Maximum-likelihood tree of all full-length amino acid sequences of EnvP(b)1 ORFs identified from publicly available databases. Sequences in italics are newly identified in this work. Sequences highlighted in red were used for further experiments.

The EnvP(b)1 ORF has been identified previously in the *RIN3* locus of select ape and Old World monkey genomes (5). We surveyed primate genomes in publicly available databases for the EnvP(b)1 ORF. We identified 35 orthologs in simian species (New and Old World monkeys and apes). The presence of each within the *RIN3* locus was confirmed by mapping flanking sequences outside the ERV locus. The phylogenetic relationships between the simian EnvP(b)1s generally follow the same clustering as their hosts (Fig. 2). This pattern of branching is consistent with insertion of an ancestral ERVP(b)1 into the *RIN3* locus of a common ancestor of all extant simian primates, followed by divergence of the Env sequences together with their respective host species. Based on the reported divergence times of New World and Old World monkey lineages (9–11), the EnvP(b)1 insertion arose at least 40 to 47 MYA, considerably earlier than previous synteny-based estimates (5).

We were unable to identify the EnvP(b)1 ORF in prosimian lineages (tarsiers, lemurs, and lorisoids). We were also unable to find evidence of the ERV (or solo long terminal repeat) in prosimian genome assemblies of the *RIN3* locus. These observations suggest that the EnvP(b)1 insertion in simians occurred after their divergence from prosimians and before the radiation of extant simian species. Bound by these evolutionary events, the insertion of EnvP(b)1 occurred between 64 to 71 MYA and 40 to 47 MYA (Fig. S2A).

10.1128/mBio.02772-20.2 FIG S2 History of ERV-P(b) integration events. (A) Primate phylogeny highlighting the integration that gave rise to ERV-P(b)1. Branches highlighted in solid red lines indicate lineages in which ERV-P(b)1 is present. The blue box denotes the branch corresponding to the most recent possible integration of ERV-P(b)1. The dashed red branch indicates that related sequences have been identified among aye-aye genomic sequencing reads. The tree is an illustrative representation of data presented in reference 9. (B) Reads from deposited aye-aye whole-genome shotgun sequencing mapped across ERV-P(b)1 from the *RIN3* locus (GenBank accession numbers AGTM011455045, AGTM012725663, AGTM010332352.1, AGTM011595709.1, AGTM012840819.1, AGTM011264183.1, AGTM012908686, AGTM010255092, AGTM010981769, AGTM011358766, and AGTM010685344). Reads colored green and cyan map to the EnvP(b)1 iRBD. (C) Aye-aye sequences mapped onto the human EnvP(b)1 iRBD structure. Colors correspond to those in panel B. Download FIG S2, TIF file, 0.6 MB. Copyright © 2020 McCarthy et al. 2020 McCarthy et al. This content is distributed under the terms of the Creative Commons Attribution 4.0 International license.

EnvP(b)1-related sequences, of any length or coding capacity, were also absent in all but one prosimian species, aye-ayes. We identified a number of short reads from an incomplete genome, which were similar to EnvP(b)1 and the surrounding ERV (Fig. S2B). These could not be unambiguously assigned to a specific ERV-P(b) locus. How these sequences entered the aye-aye genome is unknown. Aye-ayes diverged from other extant lemurs ∼55 MYA (9–12), shortly after lemurs colonized Madagascar and became geographically isolated between 55 and 75 MYA (13, 14). EnvP(b)1-related sequences were also absent in all publicly available databases of mammalian, avian, reptilian, amphibian, piscine, and insect genomes.

### EnvP(b)1 orthologs fuse cells of diverse tissue and species origin.

**FIG 3:**
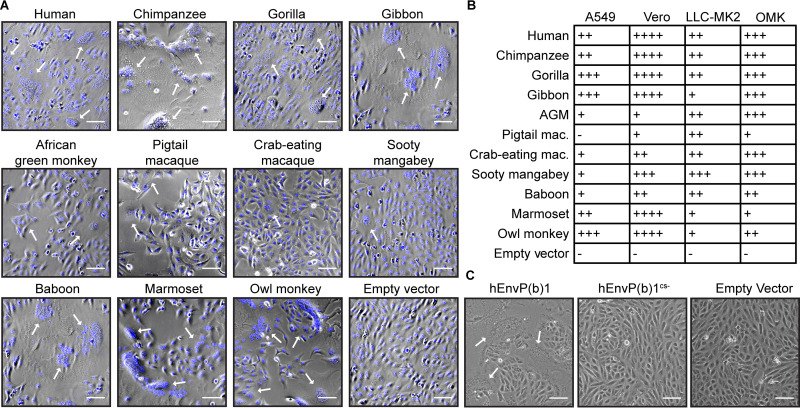
EnvP(b)1 fusogenicity has been maintained over the course of simian evolution. (A) Representative fusion assays performed in Vero (green monkey) cells. Cells were transfected with EnvP(b)1 ORFs from the indicated species, allowed to fuse, fixed, and later stained with nuclear stain (blue) prior to imaging. Arrows indicate multinucleated syncytia arising from EnvP(b)1-mediated cell fusion. Bar, 100 μm. (B) Summary of the relative fusogenicity of simian EnvP(b)1 orthologs expressed in cells from divergent simian species. Fusogenicity of each EnvP(b)1 in each cell was determined by approximating the percentage of total cells fused under each condition. +, 1 to 25%; ++, 26 to 50%; +++, 51 to 75%; ++++, 75 to 100%. Cell line species: A549, human (ape); Vero, green monkey (Old World monkey); LLC-MK2, macaque (Old World monkey); OMK, owl monkey (New World monkey). AGM, African green monkey; Mac, macaque. (C) A furin cleavage site mutant does not fuse cells. Vero cells were transfected with human EnvP(b)1, human EnvP(b)1^cs−^, or empty vector. Arrows indicate syncytia. Bar, 100 μm.

EnvP(b)1 has evolved under at least three consecutive stages comprising distinctly different selective pressures: (i) as the Env protein of a circulating virus prior to endogenization, 40 to 71 MYA; (ii) as a captured ERV during the process of cooption by the host; and (iii) as a gene under purifying selection in all simian species. The fact that the EnvP(b)1 ORF has been retained throughout simian evolution suggests that it may perform a host function(s). Expression of human EnvP(b)1 has been shown to drive the formation of multinucleated syncytia in cultured cells, an indication of cell-cell fusion (7, 8). We examined the expression, proteolytic processing, and ability to mediate cell-cell fusion of 11 divergent simian EnvP(b)1 orthologs that were expressed from cells of human, Old World monkey, and New World monkey origin. All EnvP(b)1s were expressed in all cell lines, as shown by Western blotting (Fig. S3). Similar to our observations in 293T cells, and unlike in human tissue lysate, the dominant form of the protein was unprocessed SU-TM. EnvP(b)1 orthologs from New World monkeys (marmoset and owl monkey) were detected at lower levels than those from apes and Old World monkeys, possibly due to sequence differences in the iRBD compared to the human ortholog (against which the serum was raised). All 11 EnvP(b)1 orthologs mediated cell-cell fusion in each of the cell lines tested, with the exception of the pigtail macaque ortholog in human A549 cells (Fig. 3A and B). Fusion efficiency varied between EnvP(b)1 orthologs and cell lines (Fig. 3B and Fig. S4). EnvP(b)1 from great ape species (human, chimpanzee, and gorilla) were generally the most fusogenic across all cell lines. In these assays, the extent of syncytium formation did not correlate with the extent of apparent EnvP(b)1 expression or processing. Proteolytic processing is essential for fusion, as we observed complete loss of fusogenicity with a variant of the human EnvP(b)1 ortholog in which the predicted furin cleavage site was mutated (Fig. 3C).

10.1128/mBio.02772-20.3 FIG S3 Expression of EnvP(b)1 from multiple primates in primate cell lines. Cell lysates from the indicated cell lines transfected with the EnvP(b)1 from the indicated species were subjected to Western blotting with either anti-EnvP(b)1 serum or anti-GAPDH antibody. Download FIG S3, TIF file, 1.9 MB. Copyright © 2020 McCarthy et al. 2020 McCarthy et al. This content is distributed under the terms of the Creative Commons Attribution 4.0 International license.

10.1128/mBio.02772-20.4 FIG S4 Preservation of fusogenicity of EnvP(b)1. (A) Examples of cell fusion results. The indicated cell lines were transfected with EnvP(b)1 ORFs from the indicated species. Cells were stained with nuclear stain (blue) and imaged. Bar, 100 μm. Cell line species: A549, human; Vero, green monkey; LLC-MK2, macaque; OMK, owl monkey. Download FIG S4, TIF file, 2.6 MB. Copyright © 2020 McCarthy et al. 2020 McCarthy et al. This content is distributed under the terms of the Creative Commons Attribution 4.0 International license.

### Structure of the EnvP(b)1 inferred receptor binding domain.

**FIG 4:**
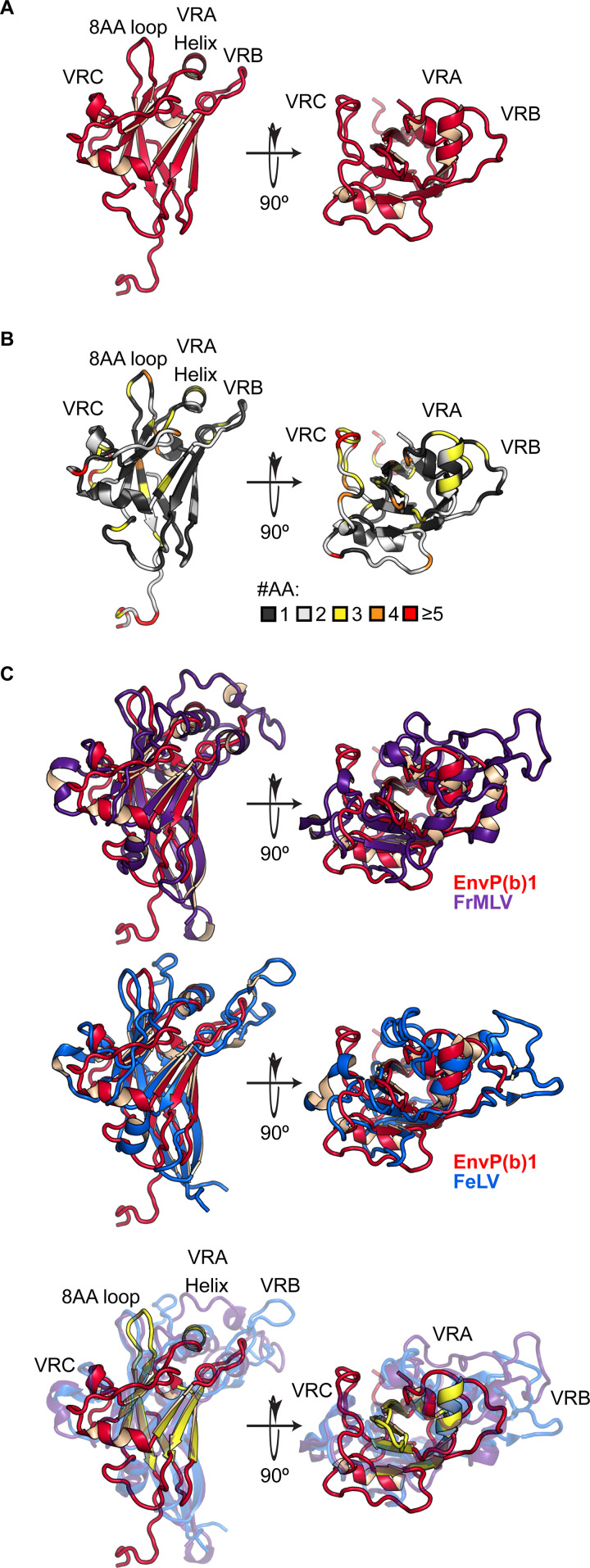
Structure of the EnvP(b)1 inferred receptor binding domain (iRBD). (A) Crystal structure of EnvP(b)1 iRBD. A C-terminal tag that is present in the structure was removed in this representation. A single glycan, resolved in the structure, is also not shown. Variable regions (VRA, VRB, and VRC), the 8-amino-acid loop, and the central helix are annotated. (B) Amino acid diversity of the simian EnvP(b)1 iRBD. Amino acid diversity was compared between 35 simian EnvP(b)1 iRBD sequences. Each residue is colored based on the number of unique amino acids found at that position. (C) Comparison of EnvP(b)1 iRBD with RBD structures of extant gammaretroviruses. (Top) EnvP(b)1 (red) overlaid with FrMLV (purple) (PDB code 1AOL). (Middle) EnvP(b)1 overlaid with FeLV (blue) (PDB code 1LCS). (Bottom) Overlay of all three RBD structures. Common features of the beta sheet core, central helix, and 8-amino-acid loop are highlighted in yellow on the EnvP(b)1 structure.

We determined the crystal structure of the human EnvP(b)1 iRBD. Single anomalous dispersion (SAD) from a platinum derivative was used to obtain experimental phases and ultimately produce a model (Fig. 4A). The iRBD’s core is defined by a β sandwich that shares some similarity with a V-type Ig fold, although it lacks the first β sheet at the N terminus. Computational analysis suggests that the rest of the N-terminal region of EnvP(b)1 is helical and that it is cleaved by signal peptidase. Extensions between the β sheets form a series of loops and α-helical regions that decorate the core. There is weak sequence identity between these features and the gammaretrovirus variable regions A, B, and C (VRA, VRB, and VRC, respectively), which are believed to dictate Env-receptor interactions for gammaretroviruses (15, 16).

The iRBD is conserved in sequence among the 35 simian EnvP(b)1 orthologs (Fig. S5), with insertions and deletions present only in the unstructured C terminus. We mapped amino acid diversity to the iRBD structure by scoring the number of different amino acids that were present at each position (Fig. 4B). For the majority of the domain, only one or two amino acids are present at a given site. Substitutions within the core were often conservative. Most of the sites that tolerate amino acid diversity are in loops or surface exposed residues on helices. In particular, the sequence of VRC has diverged the most rapidly, suggesting that its sequence is not subject to the same constraints as other regions of the iRBD.

10.1128/mBio.02772-20.5 FIG S5 Alignment of iRBD amino acid sequence from 35 simian species. Variable regions VRA, VRB, and VRC are annotated. This alignment was used to determine the amino acid variability at each site, shown in Fig. 3B. Download FIG S5, TIF file, 0.6 MB. Copyright © 2020 McCarthy et al. 2020 McCarthy et al. This content is distributed under the terms of the Creative Commons Attribution 4.0 International license.

We compared the iRBD structure to the two other known gammaretrovirus RBD structures. These are from Friend murine leukemia virus (FrMLV) and feline leukemia virus (FeLV), both extant circulating viruses (15, 17). The iRBD shares little (∼20%) sequence identity with these examples, yet it shows extremely strong structural similarities. The EnvP(b)1 iRBD is more compact with shorter, less prominent loop structures extending from its core (Fig. 4C). All three VRs are shorter in EnvP(b)1: VRA and VRB are dramatically shorter, and the VRC, also shorter, adopts a different conformation as it is liberated from constraints by the absence of the paired Cys residues that form a disulfide in FrMLV and FeLV. Additional connecting regions also exhibit changes in length and corresponding structural differences. As a consequence, the potential receptor-interacting interfaces of EnvP(b)1 are quite different from those for FrMLV and FeLV. However, there is clearly a structurally conserved architecture that is common among gammaretroviruses (Fig. S6). The β sandwich core, a central helix that runs perpendicular to it, and an 8-amino-acid loop between the last two β sheets are all structurally conserved. This conserved scaffold structure serves as a platform for the presentation of unique elements that determine receptor binding specificity.

10.1128/mBio.02772-20.6 FIG S6 Gammaretroviral RBDs share a conserved core architecture. Topology diagrams of EnvP(b)1, Fr-MLV (PDB code 1AOL), and FeLV (PDB code 1LCS) receptor binding domains are shown. N and C termini are shown as boxes. β sheets are depicted as arrows and α helices as cylinders. Conserved sheets and helices are colored yellow and numbered, and unique elements are colored red. In Fr-MLV, a white box with a dashed outline is used to denote a region with β sheet-like geometry that corresponds to a conserved β sheet in EnvP(b)1 and FeLV. Conserved disulfide bonds are shown as dark blue lines, and unique disulfide bonds are shown in light blue. The Dali protein structure comparison server (40) was used to determine percent identity (%ID), root mean square deviation (RMSD), and DALI Z-scores for these three proteins. Download FIG S6, TIF file, 0.3 MB. Copyright © 2020 McCarthy et al. 2020 McCarthy et al. This content is distributed under the terms of the Creative Commons Attribution 4.0 International license.

## DISCUSSION

Captured viral diversity has produced host genetic novelty. Viral Env proteins have at least two activities: receptor binding and membrane fusion. In cells, Env-receptor interactions can result in receptor blockade or downregulation of the receptor from the cell surface. Receptor engagement is also often a trigger that potentiates membrane fusion. Over the course of animal evolution, these viral functions have been coopted in multiple instances to perform host functions. ERV Envs that have been coopted are diverse in their ages, sequences, and, where known, binding partners (18). It appears that capturing and maintaining a variety of genes from divergent viruses provides evolutionary benefits.

EnvP(b)1 has all the hallmarks of a viral fusogen that has been repurposed to perform a host function. In contrast to other ERV-P(b)1 genes, the EnvP(b)1 ORF has been maintained as a coopted gene for the entirety of simian evolution, and likely longer. At least one of its functions, membrane fusion, has also been retained in every simian clade. Syncytin-2 (ERV-FRD Env), which is thought to play a key role in the formation of the syncytiotrophoblast layer of the placenta, is the only other ERV Env known to have fusogenic members in all simian clades (19–21). Our transfection and fusion experiments, performed in cells derived from divergent primate species, indicate that the unidentified cellular factors required for fusion, such as receptors and proteases, are also conserved. In these assays, ape and New World monkey EnvP(b)1 orthologs tended to behave more similarly to each other, while Old World monkey EnvP(b)1s were overall less fusogenic. These lineage-specific differences may reflect the consequences of divergence and coevolution of EnvP(b)1 orthologs with the factors that are required for cell fusion.

EnvP(b)1 protein is expressed in multiple healthy human tissues. In these tissues, it exists in a processed, presumably fusion-competent form. We detected the fully processed SU form and no unprocessed SU-TM in all EnvP(b)1-expressing tissues. In contrast, SU-TM was the major species in transfected cells. We speculate that in tissues, expression of EnvP(b)1 and the proper furin-like protease may be coordinated. Under nonreducing conditions, EnvP(b)1 is predominantly found in higher-molecular-weight species in both tissues and transfected cells, similar to oligomeric species observed for syncytin-1 (ERV-W Env) under nonreducing conditions (22, 23), indicating that disulfide-linked and fusion-primed SU-TM is present in these tissues. Our detection of protein was generally in agreement with the previously reported RNA abundances, with the exception of splenic tissue, where we observed only a band of a different molecular weight (5, 7). Placental tissue had the highest expression of EnvP(b)1 protein. In other tissues, expression did not vary with pregnancy or gender. The function of EnvP(b)1 is unknown, but its maintenance among simians for at least 40 to 71 million years and its expression and processing in human tissues indicate that it must in some way contribute to an important physiological process, likely involving membrane fusion.

Among human examples, selective preservation or loss of furin processing has accompanied cooption. The two syncytins are activated by furin-like cleavage events and act as membrane fusogens (2). HERV-T is no longer processed, is not fusogenic, and has evolved to downregulate its receptor (24). HEMO, of unknown function, is not classically processed by proteases but has evolved to be shed from cells via an alternative cleavage event (25). Others, like recently integrated HERV-K copies, may have been too recently acquired to have gained or lost functions. EnvP(b)1 has clearly evolved to retain its processing and fusogenicity.

EnvP(b)1 and the original infectious progenitor virus are quite ancient. We have determined that the EnvP(b)1 ORF has been maintained within the *RIN3* locus for the duration of simian primate evolution. Insertion into this locus occurred after the divergence of simians from prosimians and before the radiation of extant simian species, or between 71 and 40 million years ago. One study, using LTR dating approaches, has estimated ERV-P(b)1 to be as old as 55 million years (6). We identified a number of related sequences in the genome of an aye-aye, a species which has evolved in isolation on Madagascar for the past 55 to 75 million years (13, 14). Due to their short length and an incomplete aye-aye genome, we cannot resolve the relationship of these sequences with EnvP(b)1 or other related loci in simian genomes. Nonetheless, it appears likely that interactions between primates and related circulating virus are ancient and were widely distributed.

EnvP(b)1 has been preserved in simian genomes for at least 40 to 71 million years of evolution. We determined a structure of the iRBD from this ancient viral element. The iRBD amino acid sequence is largely conserved among the EnvP(b)1 loci found in host genomes. Though the EnvP(b)1 ORF as a whole is under purifying selection, some diversity has accumulated in the variable regions. These sites evolve rapidly in circulating viruses, and their divergence in EnvP(b)1 indicates that such sites can tolerate substitution without compromising function even in the context of a coopted protein. This conservation and the lack of insertions/deletions suggest that the current iRBD may closely approximate that of the infectious progenitor virus that circulated 40 to 71 MYA.

Comparisons with RBD structures from two extant gammaretroviruses highlight the exquisite evolutionary plasticity of this domain. All three share a conserved architecture that depends little on sequence conservation. Upon this common scaffold, each Env has evolved a series of unique features. EnvP(b)1 is the most compact, while the cores of FeLV and Fr-MLV are decorated by large structured extensions. The lack of sequence conservation prevents us from tracing the origins of these features. Whether the elaborate elements reflect a consequence of ongoing virus-host evolution (e.g., antibody escape or receptor evolution) over timescales greatly exceeding 40 to 71 million years or whether contemporary Envs retain RBD domains that are equivalently compact can only be resolved through additional structures. It does, however, underscore the versatility of this fold as a platform for recognizing many different receptor molecules. This versatility in receptor recognition may underlie the evolutionary success of gammaretroviruses, and of the Env proteins themselves, as evidenced by their wide distribution as viral fusogens, ERV elements, and coopted genes.

## MATERIALS AND METHODS

### Cell lines.

Human 293F cells were maintained at 37°C with 5% CO_2_ in FreeStyle 293 expression medium (Thermo Fisher) supplemented with penicillin and streptomycin. Human 293T (ATCC CRL-3216; American Type Culture Collection), green monkey Vero (ATCC CCL-81), and rhesus macaque LLC-MK2 (ATCC CCL-7) cells were grown in Dulbecco’s modified Eagle’s medium (DMEM) supplemented with 10% fetal bovine serum (FBS) and maintained at 37°C and 5% CO_2_. Owl monkey OMK cells (ATCC CRL-1556) were grown in Eagle’s minimum essential medium (EMEM) supplemented with 10% FBS and maintained at 37°C and 5% CO_2_.

### Identification of EnvP(b)1 sequences.

Human, chimpanzee, white-cheeked gibbon, African green monkey, and pigtail macaque sequences were previously published (5). All other full-length EnvP(b)1 ORFs were identified through querying publicly available databases, including NCBI GenBank, Ensmbl, and the UCSC genome browser, using the full-length human EnvP(b)1 nucleotide sequence. Databases queried included fully assembled genomes, NCBI nucleotide database, and whole-genome shotgun reads identified through the basic local alignment tool (BLAST). Details of these sequences are shown in Table S2.

10.1128/mBio.02772-20.8 TABLE S2 Sequence information for simian EnvP(b)1 ORFs. Loci reference nucleotide sequences. EnvP(b)1 proteins from species highlighted in red were used for expression and fusion experiments. Download Table S2, PDF file, 0.1 MB. Copyright © 2020 McCarthy et al. 2020 McCarthy et al. This content is distributed under the terms of the Creative Commons Attribution 4.0 International license.

Sequences mapping to the ERV-P(b) provirus in the aye-aye genome were identified through BLAST using either the SU domain of human EnvP(b)1 or the entire human ERV-P(b)1 locus, querying whole-genome shotgun sequences from Daubentonia madagascariensis (aye-aye). Eleven reads were identified that mapped to ERV-P(b) from an unassembled aye-aye genome (26). Accession numbers for these reads are AGTM011455045, AGTM012725663, AGTM010332352.1, AGTM011595709.1, AGTM012840819.1, AGTM011264183.1, AGTM012908686, AGTM010255092, AGTM010981769, AGTM011358766, and AGTM010685344.

### Plasmids.

Human EnvP(b)1 codon-optimized sequence was ordered from GenScript. EnvP(b)1 sequences from chimpanzee, white-cheeked gibbon, gorilla, African green monkey, pigtail macaque, crab-eating macaque, sooty mangabey, baboon, common marmoset, and owl monkey were ordered from Genewiz. All sequences were cloned into a modified version of pVRC-8400 expression vector (27). EnvP(b)1^CS−^ with the furin cleavage site mutated from RKTR to SKTR was generated from the human EnvP(b)1 plasmid using QuikChange site-directed mutagenesis (Agilent). EnvP(b)1 iRBD sequences were synthesized and cloned into a modified pVRC8400 expression vector, as previously described (28, 29).

### Recombinant EnvP(b)1 iRBD expression and purification.

The inferred receptor binding domain, residues 51 to 210, of human EnvP(b)1, was deduced as follows. A region of homology to murine and feline leukemia virus RBDs was identified using the Phyre2 server (30). The signal peptide cleavage site was predicted using the SignalP-4.1 server (31) and Phyre2 secondary structure prediction (30). This defined the iRBD N terminus. The C-terminal boundary was defined by the transition from a predicted unstructured region to the proline-rich region. iRBDs were synthesized and cloned into a modified pVRC8400 mammalian expression vector. We produced a variant with a C-terminal human rhinovirus 3C protease cleavage site followed by a 6×His tag and a variant with the following C-terminal tag configuration: an HA-tag followed by a rhinovirus 3C protease cleavage site, an 8×His tag, and a 6×His tag. Short Gly-Gly-Ala linkers separate the HA, 3C, and poly-His tags. iRBD variants were produced by polyethylenimine (PEI)-facilitated transient transfection of 293F cells that were maintained in FreeStyle 293 expression medium. Transfection complexes were prepared in Opti-MEM and added to cells. Supernatants were harvested 4 to 5 days posttransfection and clarified by low-speed centrifugation. iRBDs were purified by passage over cobalt-nitrilotriacetic acid (Co-NTA) agarose (Clontech) followed by gel filtration chromatography on Superdex 200 (GE Healthcare) in 10 mM Tris-HCl, 150 mM NaCl at pH 7.5 (buffer A).

For immunization of rabbits and production of antisera enriched for iRBD-directed IgGs, we used the version with the 3C protease site and 6×His tag. Both were removed by treatment with PreScission protease (MolBioTech; ThermoScientific). The protein was repurified on Co-NTA agarose followed by gel filtration chromatography on Superdex 200 (GE Healthcare) in buffer A to remove the protease, tag, and uncleaved protein. Crystallographic studies used the hemagglutinin (HA)-tagged version, which was not treated with protease to remove its poly-His tags.

### Generation and enrichment of EnvP(b)1 iRBD-specific antisera.

Bulk anti-iRBD serum was generated by Covance. Purified recombinant EnvP(b)1 iRBD was used to immunize a single rabbit. Serum from the terminal bleed was tested for detection of EnvP(b)1 by Western blot. Crude rabbit serum was diluted 1:50 in buffer A. Bulk IgGs were captured by protein A agarose resin (GoldBio) at 4°C with rotating overnight. The resin was collected in a chromatography column, washed with a column volume of buffer A, and eluted with 0.1 M glycine buffer (pH 2.5), which was immediately neutralized by 1 M Tris (pH 8.0). Antibodies were then dialyzed against phosphate-buffered saline (PBS) (pH 7.4). To produce an EnvP(b)1 iRBD affinity resin, EnvP(b)1 was expressed and purified as described above. Cleaved iRBD was then concentrated, dialyzed against phosphate-buffered saline (PBS) (pH 7.4), and arrested to Pierce *N*-hydroxysuccinimide (NHS)-activated agarose beads per the manufacturer’s protocol. The reaction was then quenched by the addition of Tris (pH 7.5). Rabbit antibodies were then incubated with the iRBD resin at 4°C with rotating overnight. The iRBD resin was collected in a chromatography column, washed with a column volume of PBS (pH 7.4), and eluted with 0.1 M glycine buffer (pH 2.5), which was immediately neutralized by 1 M Tris (pH 8.0). Antibodies were then dialyzed against PBS (pH 7.4).

### Crystallization, structure determination, and refinement.

EnvP(b)1 iRBD, with its one native N-linked glycan and a C-terminal tag, was concentrated to 10 to 12 mg/ml. Crystals were grown in hanging drops over a reservoir of 0.5 M sodium chloride, 0.1 M bis-Tris propane (pH 7.0), 20% (wt/vol) polyethylene glycol 4000. Platinum was incorporated by incubating crystals in a solution of 0.08 M bis-Tris propane, 0.4 M sodium formate, 16% (wt/vol) polyethylene glycol 4000, and 10 mM potassium tetrachloroplatinate(II) for approximately 48 h. All crystals were cryoprotected in 22 to 25% (vol/vol) glycerol. We recorded diffraction data at the Advanced Light Source on beamline 8.2.2 and on the Advanced Photon Source on beamlines 24-ID-E and 24-ID-C.

Using XDS (32), data were processed for one native crystal and the single anomalous dispersion (SAD) data were obtained for one platinum derivative crystal. The merged data used for phasing had an anomalous signal to about 3.5 Å resolution. Using SHELX (33), we found 5 platinum sites, refined them, and calculated phase probabilities. Initial maps were obtained after statistical density modification using RESOLVE (34). The Matthew coefficient suggested that there were multiple iRBD copies in the asymmetric unit. We flooded the initial map with dummy atoms and carved out a single copy of iRBD (the one with the best-resolved density) by inspection in PyMol (Schrödinger, LLC). With this we obtained a search model to locate two additional copies of iRBD in the asymmetric unit by molecular replacement using PHASER (35). After further optimizing the phases by 3-fold noncrystallographic symmetry (NCS) averaging, we obtained a map of reasonable quality to build an initial model that was then used to phase the native data set.

We carried out refinement calculations with PHENIX (36) and model modifications with COOT (37). Refinement of atomic positions and B factors was followed by translation-liberation-screw (TLS) parameterization and placement of water molecules. Final coordinates were validated with the MolProbity server (38). Data collection and refinement statistics are in Table S3. Figures were made with PyMol.

10.1128/mBio.02772-20.9 TABLE S3 Crystallographic statistics for EnvP(b)1 iRBD. Download Table S3, PDF file, 0.1 MB. Copyright © 2020 McCarthy et al. 2020 McCarthy et al. This content is distributed under the terms of the Creative Commons Attribution 4.0 International license.

### Comparison of RBD structures.

Topology diagrams of the EnvP(b)1 iRBD, Friend murine leukemia virus RBD (PDB code 1LCS) (15), and feline leukemia virus RBD (PDB code 1AOL) (17) were modified from those created by PDBSum (39). We used the Dali protein structure comparison server (40) to determine the percent identity, root mean square deviation (RMSD), and DALI Z-scores for these three proteins.

### Human tissue lysates and cell lysate Western blots.

Tissue protein lysates were purchased from TaKaRa. Details for each lysate are shown in Table S1. Tissue lysate was mixed with Laemmli buffer with or without (for nonreducing conditions) 2-mercaptoethanol and boiled for 5 min. A 40-μg portion of tissue lysate was run on a 4 to 20% acrylamide gel (Bio-Rad).

Human EnvP(b)1, EnvP(b)1^cs−^, HERV-W Env, and empty vector were transfected into 293T cells using Lipofectamine 3000 reagent (Life Technologies). Human, chimpanzee, gorilla, gibbon, African green monkey, pigtail macaque, sooty mangabey, baboon, crab-eating macaque, owl monkey, and marmoset EnvP(b)1 plasmids were transfected into A549, Vero, LLC-MK2, and OMK cells using Lipofectamine 3000 reagent. At 24 h posttransfection, cells were lysed in TNE buffer (50 mM Tris, 2 mM EDTA, 150 mM NaCl) supplemented with 1% Triton X-100. Lysates were boiled in Laemmli buffer with or without (for nonreducing condition for A549 lysate) 2-mercaptoethanol and run on 4 to 20% acrylamide gels.

For all Western blots, proteins were transferred onto nitrocellulose membrane. Membranes were blocked with 5% milk in PBS–0.1% Tween 20 and probed with anti-EnvP(b)1 serum or mouse anti-GAPDH antibody (Genscript), followed by horseradish peroxidase (HRP)-conjugated goat anti-mouse immunoglobulin (Invitrogen) or HRP-conjugated goat anti-rabbit immunoglobulin (Sigma-Aldrich) antibodies. Membranes were incubated with enhanced chemiluminescence (ECL) reagent (Thermo), and signal was detected using an Amersham imager 600 device (GE Healthcare). Images were processed using ImageJ software (National Institutes of Health).

### Cell-cell fusion.

Human, chimpanzee, gorilla, gibbon, African green monkey, pigtail macaque, sooty mangabey, baboon, crab-eating macaque, owl monkey, and marmoset EnvP(b)1 plasmids were transfected into A549, Vero, LLC-MK2, and OMK cells using Lipofectamine 3000 reagent. At 18 to 24 h posttransfection, cells were stained with NucBlue live cell nuclear stain (Life Technologies) for 20 min at 37°C and subsequently imaged. Images were processed using ImageJ software.

### Data availability.

Coordinates and diffraction data have been deposited at the Protein Data Bank (PDB code 6W5Y).

## References

[1] Lander ES, Linton LM, Birren B, Nusbaum C, Zody MC, Baldwin J, Devon K, Dewar K, Doyle M, FitzHugh W, Funke R, Gage D, Harris K, Heaford A, Howland J, Kann L, Lehoczky J, LeVine R, McEwan P, McKernan K, Meldrim J, Mesirov JP, Miranda C, Morris W, Naylor J, Raymond C, Rosetti M, Santos R, Sheridan A, Sougnez C, Stange-Thomann Y, Stojanovic N, Subramanian A, Wyman D, Rogers J, Sulston J, Ainscough R, Beck S, Bentley D, Burton J, Clee C, Carter N, Coulson A, Deadman R, Deloukas P, Dunham A, Dunham I, Durbin R, French L, Grafham D, International Human Genome Sequencing Consortium, . 2001. Initial sequencing and analysis of the human genome. Nature 409:860–921. doi:10.1038/35057062. 11237011

[2] Lavialle C, Cornelis G, Dupressoir A, Esnault C, Heidmann O, Vernochet C, Heidmann T. 2013. Paleovirology of 'syncytins', retroviral env genes exapted for a role in placentation. Philos Trans R Soc Lond B Biol Sci 368:20120507. doi:10.1098/rstb.2012.0507. 23938756 PMC3758191

[3] de Parseval N, Lazar V, Casella JF, Benit L, Heidmann T. 2003. Survey of human genes of retroviral origin: identification and transcriptome of the genes with coding capacity for complete envelope proteins. J Virol 77:10414–10422. doi:10.1128/jvi.77.19.10414-10422.2003. 12970426 PMC228468

[4] Villesen P, Aagaard L, Wiuf C, Pedersen FS. 2004. Identification of endogenous retroviral reading frames in the human genome. Retrovirology 1:32. doi:10.1186/1742-4690-1-32. 15476554 PMC524368

[5] Aagaard L, Villesen P, Kjeldbjerg AL, Pedersen FS. 2005. The approximately 30-million-year-old ERVPb1 envelope gene is evolutionarily conserved among hominoids and Old World monkeys. Genomics 86:685–691. doi:10.1016/j.ygeno.2005.08.011. 16263238

[6] Martins H, Villesen P. 2011. Improved integration time estimation of endogenous retroviruses with phylogenetic data. PLoS One 6:e14745. doi:10.1371/journal.pone.0014745. 21394200 PMC3048862

[7] Blaise S, de Parseval N, Heidmann T. 2005. Functional characterization of two newly identified human endogenous retrovirus coding envelope genes. Retrovirology 2:19. doi:10.1186/1742-4690-2-19. 15766379 PMC555746

[8] Vargas A, Thiery M, Lafond J, Barbeau B. 2012. Transcriptional and functional studies of human endogenous retrovirus envelope EnvP(b) and EnvV genes in human trophoblasts. Virology 425:1–10. doi:10.1016/j.virol.2011.12.015. 22277806

[9] Pozzi L, Hodgson JA, Burrell AS, Sterner KN, Raaum RL, Disotell TR. 2014. Primate phylogenetic relationships and divergence dates inferred from complete mitochondrial genomes. Mol Phylogenet Evol 75:165–183. doi:10.1016/j.ympev.2014.02.023. 24583291 PMC4059600

[10] Perelman P, Johnson WE, Roos C, Seuanez HN, Horvath JE, Moreira MA, Kessing B, Pontius J, Roelke M, Rumpler Y, Schneider MP, Silva A, O'Brien SJ, Pecon-Slattery J. 2011. A molecular phylogeny of living primates. PLoS Genet 7:e1001342. doi:10.1371/journal.pgen.1001342. 21436896 PMC3060065

[11] Fabre PH, Rodrigues A, Douzery EJ. 2009. Patterns of macroevolution among primates inferred from a supermatrix of mitochondrial and nuclear DNA. Mol Phylogenet Evol 53:808–825. doi:10.1016/j.ympev.2009.08.004. 19682589

[12] Pozzi L, Nekaris KA, Perkin A, Bearder SK, Pimley ER, Schulze H, Streicher U, Nadler T, Kitchener A, Zischler H, Zinner D, Roos C. 2015. Remarkable ancient divergences amongst neglected lorisiform primates. Zool J Linn Soc 175:661–674. doi:10.1111/zoj.12286. 26900177 PMC4744660

[13] Horvath JE, Weisrock DW, Embry SL, Fiorentino I, Balhoff JP, Kappeler P, Wray GA, Willard HF, Yoder AD. 2008. Development and application of a phylogenomic toolkit: resolving the evolutionary history of Madagascar's lemurs. Genome Res 18:489–499. doi:10.1101/gr.7265208. 18245770 PMC2259113

[14] Yoder AD, Yang Z. 2004. Divergence dates for Malagasy lemurs estimated from multiple gene loci: geological and evolutionary context. Mol Ecol 13:757–773. doi:10.1046/j.1365-294x.2004.02106.x. 15012754

[15] Fass D, Davey RA, Hamson CA, Kim PS, Cunningham JM, Berger JM. 1997. Structure of a murine leukemia virus receptor-binding glycoprotein at 2.0 angstrom resolution. Science 277:1662–1666. doi:10.1126/science.277.5332.1662. 9287219

[16] Battini JL, Heard JM, Danos O. 1992. Receptor choice determinants in the envelope glycoproteins of amphotropic, xenotropic, and polytropic murine leukemia viruses. J Virol 66:1468–1475. doi:10.1128/JVI.66.3.1468-1475.1992. 1310758 PMC240871

[17] Barnett AL, Wensel DL, Li W, Fass D, Cunningham JM. 2003. Structure and mechanism of a coreceptor for infection by a pathogenic feline retrovirus. J Virol 77:2717–2729. doi:10.1128/jvi.77.4.2717-2729.2003. 12552012 PMC141074

[18] Johnson WE. 2019. Origins and evolutionary consequences of ancient endogenous retroviruses. Nat Rev Microbiol 17:355–370. doi:10.1038/s41579-019-0189-2. 30962577

[19] Esnault C, Cornelis G, Heidmann O, Heidmann T. 2013. Differential evolutionary fate of an ancestral primate endogenous retrovirus envelope gene, the EnvV syncytin, captured for a function in placentation. PLoS Genet 9:e1003400. doi:10.1371/journal.pgen.1003400. 23555306 PMC3610889

[20] Blaise S, Ruggieri A, Dewannieux M, Cosset FL, Heidmann T. 2004. Identification of an envelope protein from the FRD family of human endogenous retroviruses (HERV-FRD) conferring infectivity and functional conservation among simians. J Virol 78:1050–1054. doi:10.1128/jvi.78.2.1050-1054.2004. 14694139 PMC368808

[21] Caceres M, Program NCS, Thomas JW, NISC Comparative Sequencing Program. 2006. The gene of retroviral origin syncytin 1 is specific to hominoids and is inactive in Old World monkeys. J Hered 97:100–106. doi:10.1093/jhered/esj011. 16424151

[22] Cheynet V, Ruggieri A, Oriol G, Blond JL, Boson B, Vachot L, Verrier B, Cosset FL, Mallet F. 2005. Synthesis, assembly, and processing of the Env ERVWE1/syncytin human endogenous retroviral envelope. J Virol 79:5585–5593. doi:10.1128/JVI.79.9.5585-5593.2005. 15827173 PMC1082723

[23] Roebke C, Wahl S, Laufer G, Stadelmann C, Sauter M, Mueller-Lantzsch N, Mayer J, Ruprecht K. 2010. An N-terminally truncated envelope protein encoded by a human endogenous retrovirus W locus on chromosome Xq22.3. Retrovirology 7:69. doi:10.1186/1742-4690-7-69. 20735848 PMC2936387

[24] Blanco-Melo D, Gifford RJ, Bieniasz PD. 2017. Co-option of an endogenous retrovirus envelope for host defense in hominid ancestors. Elife 6:e22519. doi:10.7554/eLife.22519. 28397686 PMC5388530

[25] Heidmann O, Beguin A, Paternina J, Berthier R, Deloger M, Bawa O, Heidmann T. 2017. HEMO, an ancestral endogenous retroviral envelope protein shed in the blood of pregnant women and expressed in pluripotent stem cells and tumors. Proc Natl Acad Sci U S A 114:E6642–E6651. doi:10.1073/pnas.1702204114. 28739914 PMC5559007

[26] Perry GH, Reeves D, Melsted P, Ratan A, Miller W, Michelini K, Louis EE, Jr, Pritchard JK, Mason CE, Gilad Y. 2012. A genome sequence resource for the aye-aye (Daubentonia madagascariensis), a nocturnal lemur from Madagascar. Genome Biol Evol 4:126–135. doi:10.1093/gbe/evr132. 22155688 PMC3273163

[27] Schmidt AG, Xu H, Khan AR, O'Donnell T, Khurana S, King LR, Manischewitz J, Golding H, Suphaphiphat P, Carfi A, Settembre EC, Dormitzer PR, Kepler TB, Zhang R, Moody MA, Haynes BF, Liao HX, Shaw DE, Harrison SC. 2013. Preconfiguration of the antigen-binding site during affinity maturation of a broadly neutralizing influenza virus antibody. Proc Natl Acad Sci U S A 110:264–269. doi:10.1073/pnas.1218256109. 23175789 PMC3538208

[28] McCarthy KR, Watanabe A, Kuraoka M, Do KT, McGee CE, Sempowski GD, Kepler TB, Schmidt AG, Kelsoe G, Harrison SC. 2018. Memory B cells that cross-react with group 1 and group 2 influenza A viruses are abundant in adult human repertoires. Immunity 48:174–184 E179. doi:10.1016/j.immuni.2017.12.009. 29343437 PMC5810956

[29] Robinson-McCarthy LR, McCarthy KR, Raaben M, Piccinotti S, Nieuwenhuis J, Stubbs SH, Bakkers MJG, Whelan SPJ. 2018. Reconstruction of the cell entry pathway of an extinct virus. PLoS Pathog 14:e1007123. doi:10.1371/journal.ppat.1007123. 30080900 PMC6095630

[30] Kelley LA, Mezulis S, Yates CM, Wass MN, Sternberg MJ. 2015. The Phyre2 web portal for protein modeling, prediction and analysis. Nat Protoc 10:845–858. doi:10.1038/nprot.2015.053. 25950237 PMC5298202

[31] Petersen TN, Brunak S, von Heijne G, Nielsen H. 2011. SignalP 4.0: discriminating signal peptides from transmembrane regions. Nat Methods 8:785–786. doi:10.1038/nmeth.1701. 21959131

[32] Kabsch W. 2010. XDS. Acta Crystallogr D Biol Crystallogr 66:125–132. doi:10.1107/S0907444909047337. 20124692 PMC2815665

[33] Sheldrick GM. 2010. Experimental phasing with SHELXC/D/E: combining chain tracing with density modification. Acta Crystallogr D Biol Crystallogr 66:479–485. doi:10.1107/S0907444909038360. 20383001 PMC2852312

[34] Terwilliger TC. 2000. Maximum-likelihood density modification. Acta Crystallogr D Biol Crystallogr 56:965–972. doi:10.1107/s0907444900005072. 10944333 PMC2792768

[35] McCoy AJ, Grosse-Kunstleve RW, Adams PD, Winn MD, Storoni LC, Read RJ. 2007. Phaser crystallographic software. J Appl Crystallogr 40:658–674. doi:10.1107/S0021889807021206. 19461840 PMC2483472

[36] Adams PD, Afonine PV, Bunkoczi G, Chen VB, Davis IW, Echols N, Headd JJ, Hung LW, Kapral GJ, Grosse-Kunstleve RW, McCoy AJ, Moriarty NW, Oeffner R, Read RJ, Richardson DC, Richardson JS, Terwilliger TC, Zwart PH. 2010. PHENIX: a comprehensive Python-based system for macromolecular structure solution. Acta Crystallogr D Biol Crystallogr 66:213–221. doi:10.1107/S0907444909052925. 20124702 PMC2815670

[37] Emsley P, Cowtan K. 2004. Coot: model-building tools for molecular graphics. Acta Crystallogr D Biol Crystallogr 60:2126–2132. doi:10.1107/S0907444904019158. 15572765

[38] Chen VB, Arendall WB, III, Headd JJ, Keedy DA, Immormino RM, Kapral GJ, Murray LW, Richardson JS, Richardson DC. 2010. MolProbity: all-atom structure validation for macromolecular crystallography. Acta Crystallogr D Biol Crystallogr 66:12–21. doi:10.1107/S0907444909042073. 20057044 PMC2803126

[39] Laskowski RA, Jabłońska J, Pravda L, Vařeková RS, Thornton JM. 2018. PDBsum: structural summaries of PDB entries. Protein Sci 27:129–134. doi:10.1002/pro.3289. 28875543 PMC5734310

[40] Holm L. 2019. Benchmarking fold detection by DaliLite v.5. Bioinformatics 35:5326–5327. doi:10.1093/bioinformatics/btz536. 31263867

