# Supplementary material for: Structure of the Receptor Binding Domain of EnvP(b)1, an Endogenous Retroviral Envelope Protein Expressed in Human Tissues
Source: mBio. 2020 Nov 17;11(6):e02772-20. doi: 10.1128/mBio.02772-20 (PMC7683403; doi:10.1128/mBio.02772-20)
Supplement: TABLE S1 [file mBio.02772-20-st001.pdf]

Table S1. Information on the human tissue lysates probed for EnvP(b)1 expression

Tissue	Tissue condition	#of donors	Sex	Age	COD	Cat #	Lot #
Placenta	Normal, whole placenta	15	F	19-33		635307	1510952A
Testis	Normal, whole testes	18	M	19-64	Truama	635309	1602008A
Ovary	Normal, whole ovary	1	F	30	Trauma	635308	1510953A
Spleen	Normal, whole spleen	1	M	55	Sudden death	635312	1404375A
Thymus	Normal	1	M	21	Sudden death	635350	1002151A
Small Intestine	Normal, whole small intestine	9	M&F	21-45	Sudden death, trauma	635311	1205370A
