# Supplementary figures and images for: Structure of the Receptor Binding Domain of EnvP(b)1, an Endogenous Retroviral Envelope Protein Expressed in Human Tissues
Source: mBio. 2020 Nov 17;11(6):e02772-20. doi: 10.1128/mBio.02772-20 (PMC7683403; doi:10.1128/mBio.02772-20)

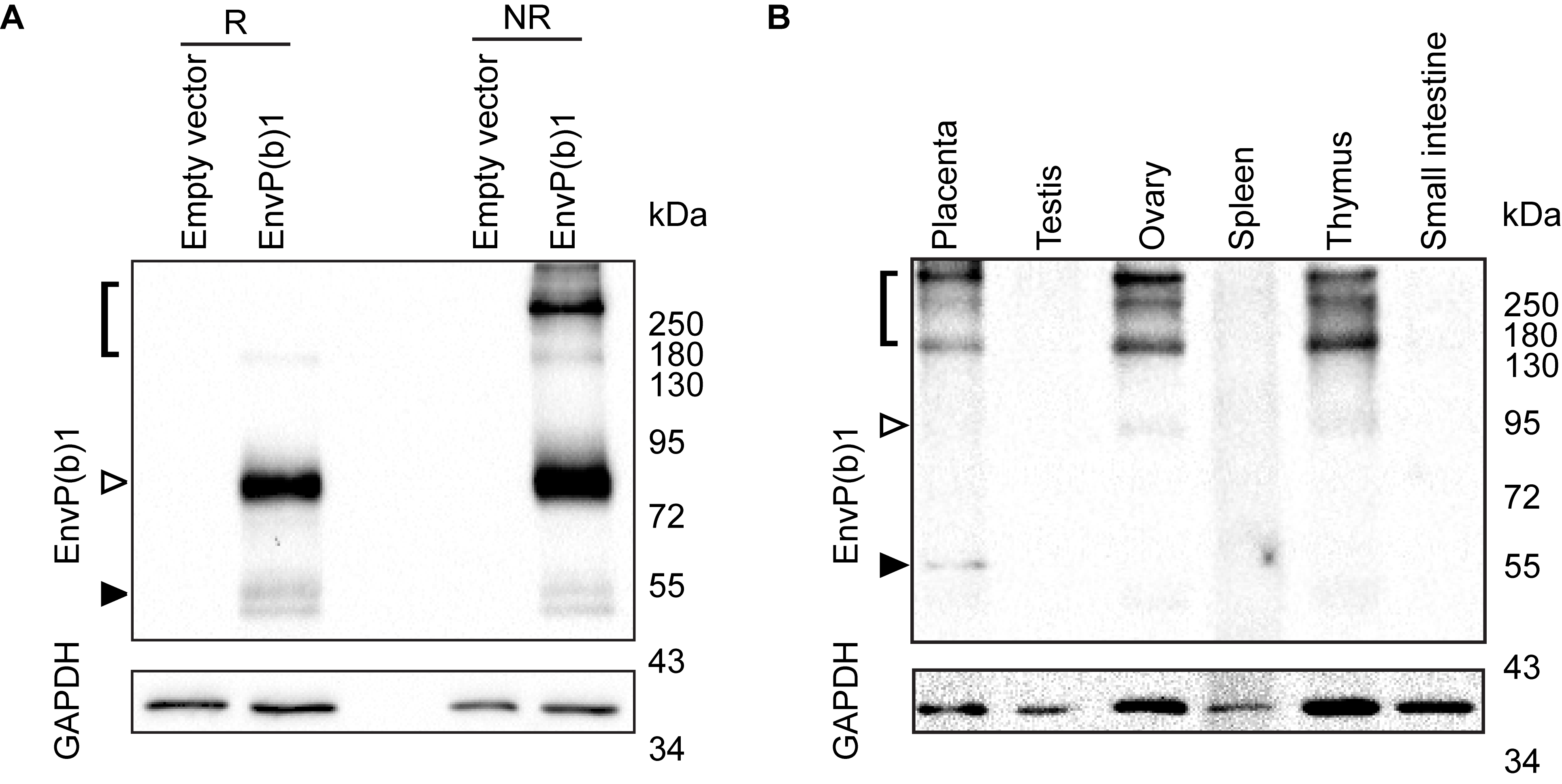

Supplement: FIG S1 [file mBio.02772-20-sf001.tif]

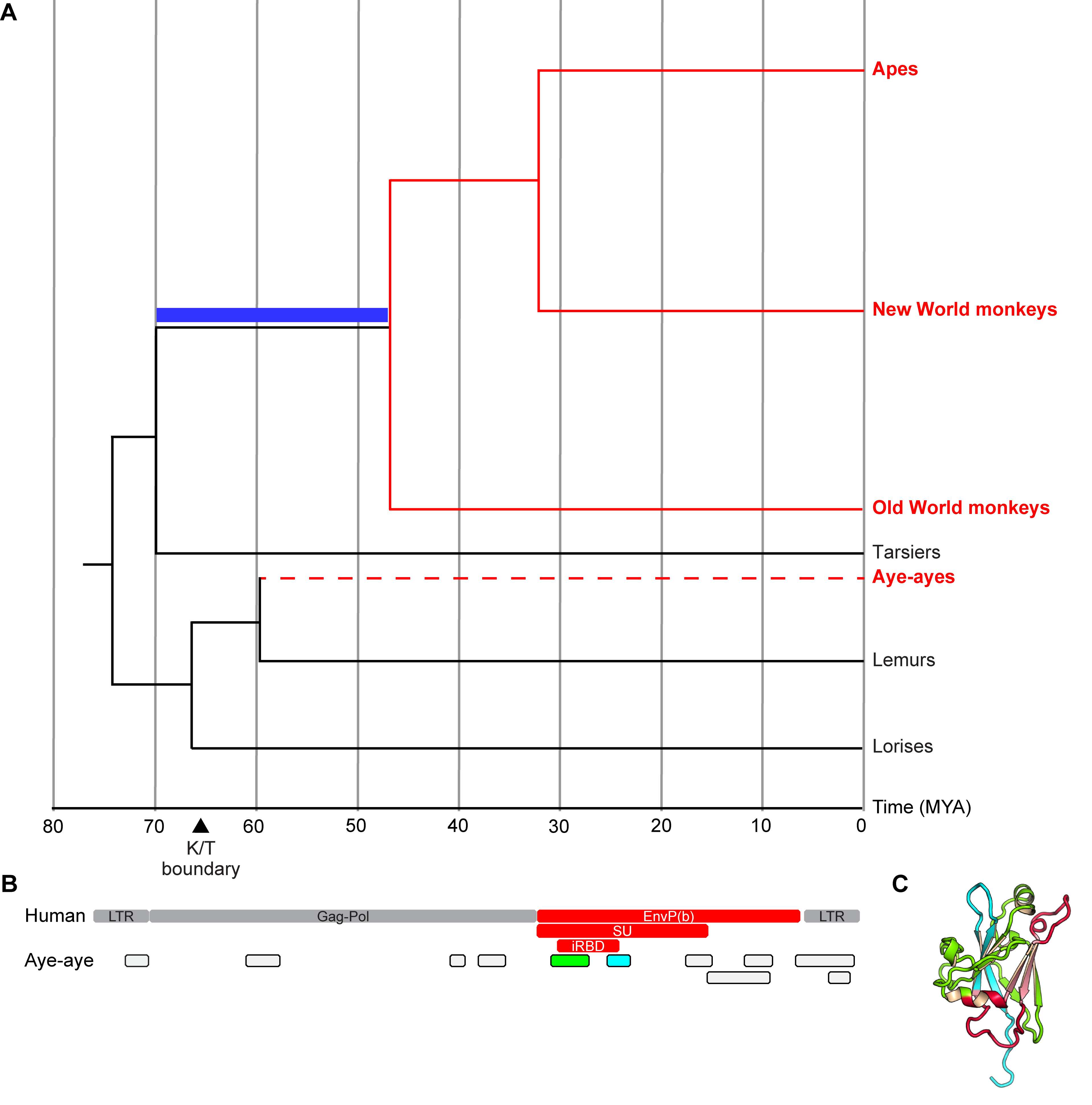

Supplement: FIG S2 [file mBio.02772-20-sf002.tif]

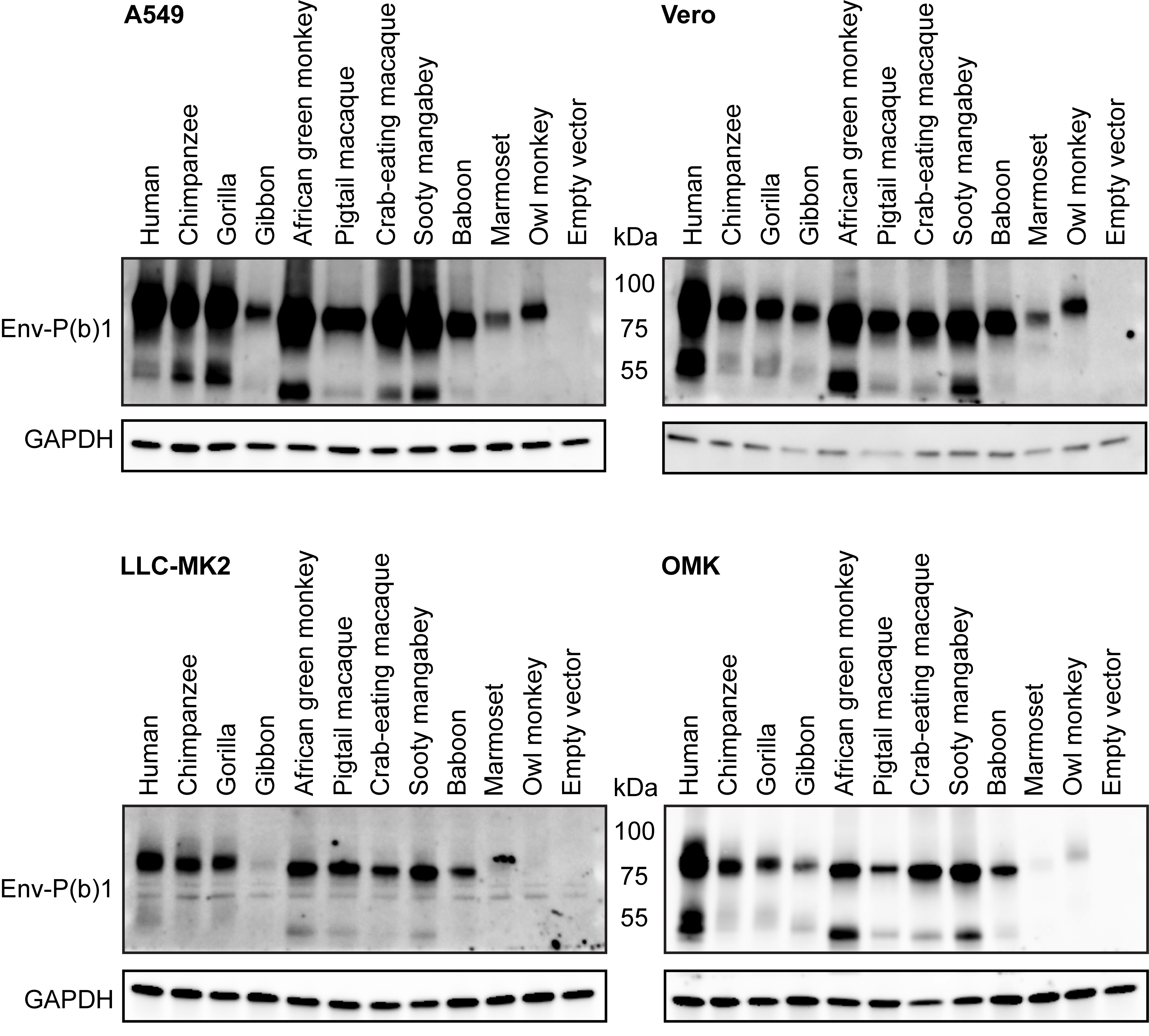

Supplement: FIG S3 [file mBio.02772-20-sf003.tif]

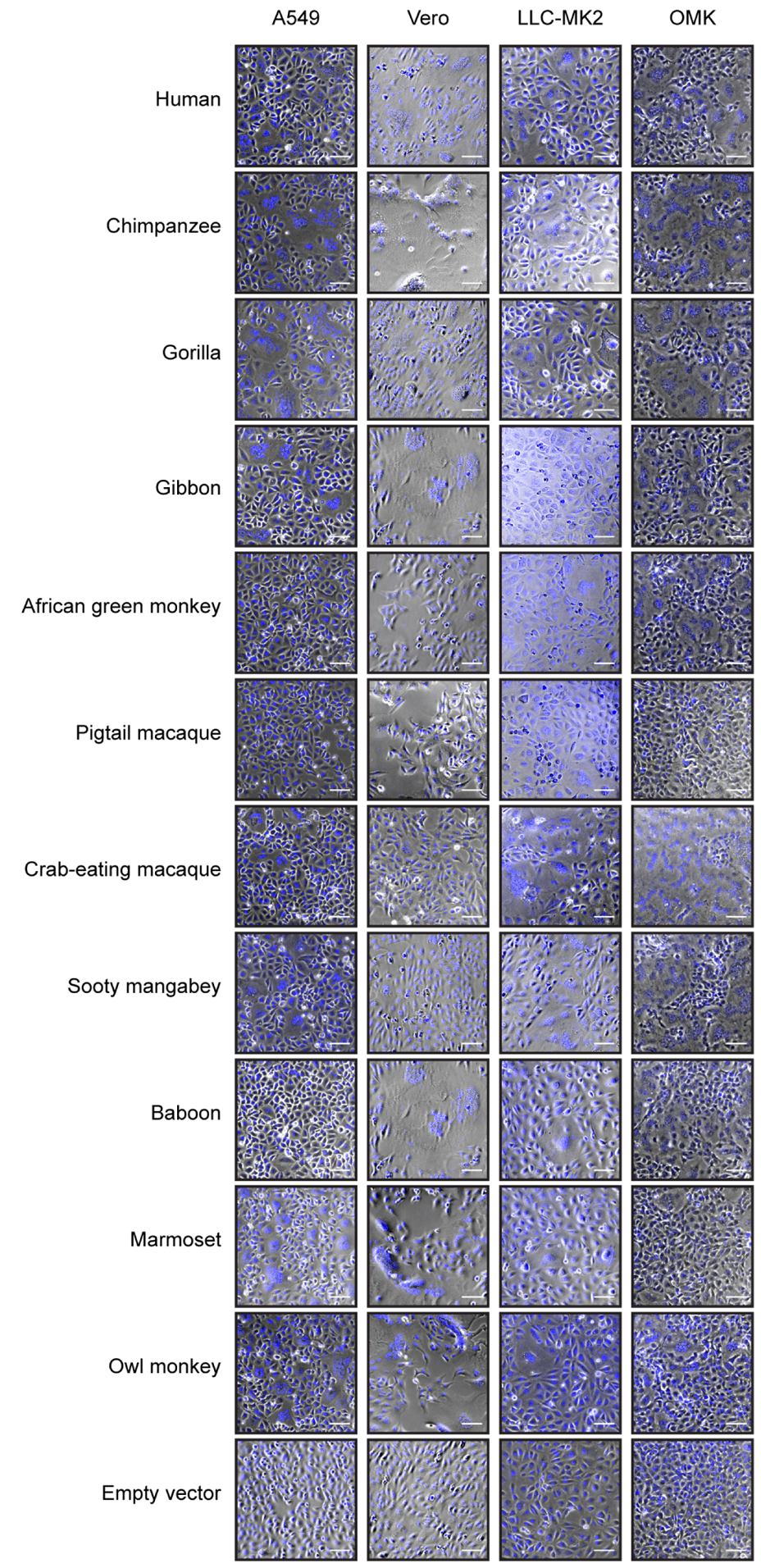

Supplement: FIG S4 [file mBio.02772-20-sf004.tif]

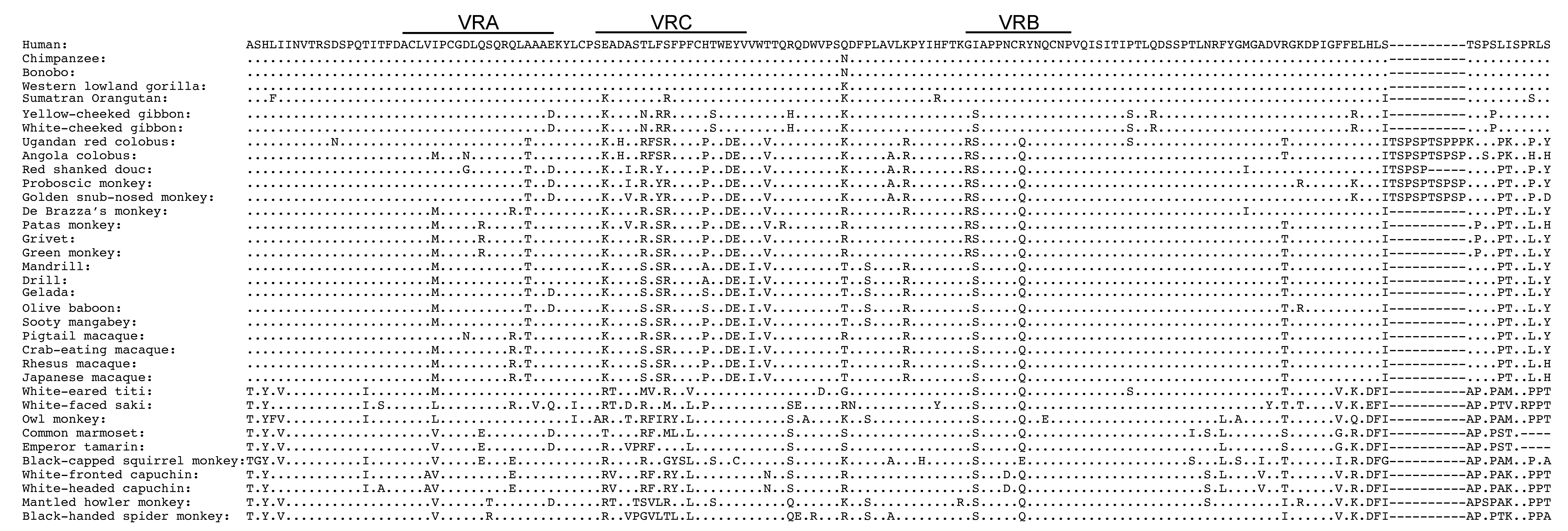

Supplement: FIG S5 [file mBio.02772-20-sf005.tif]

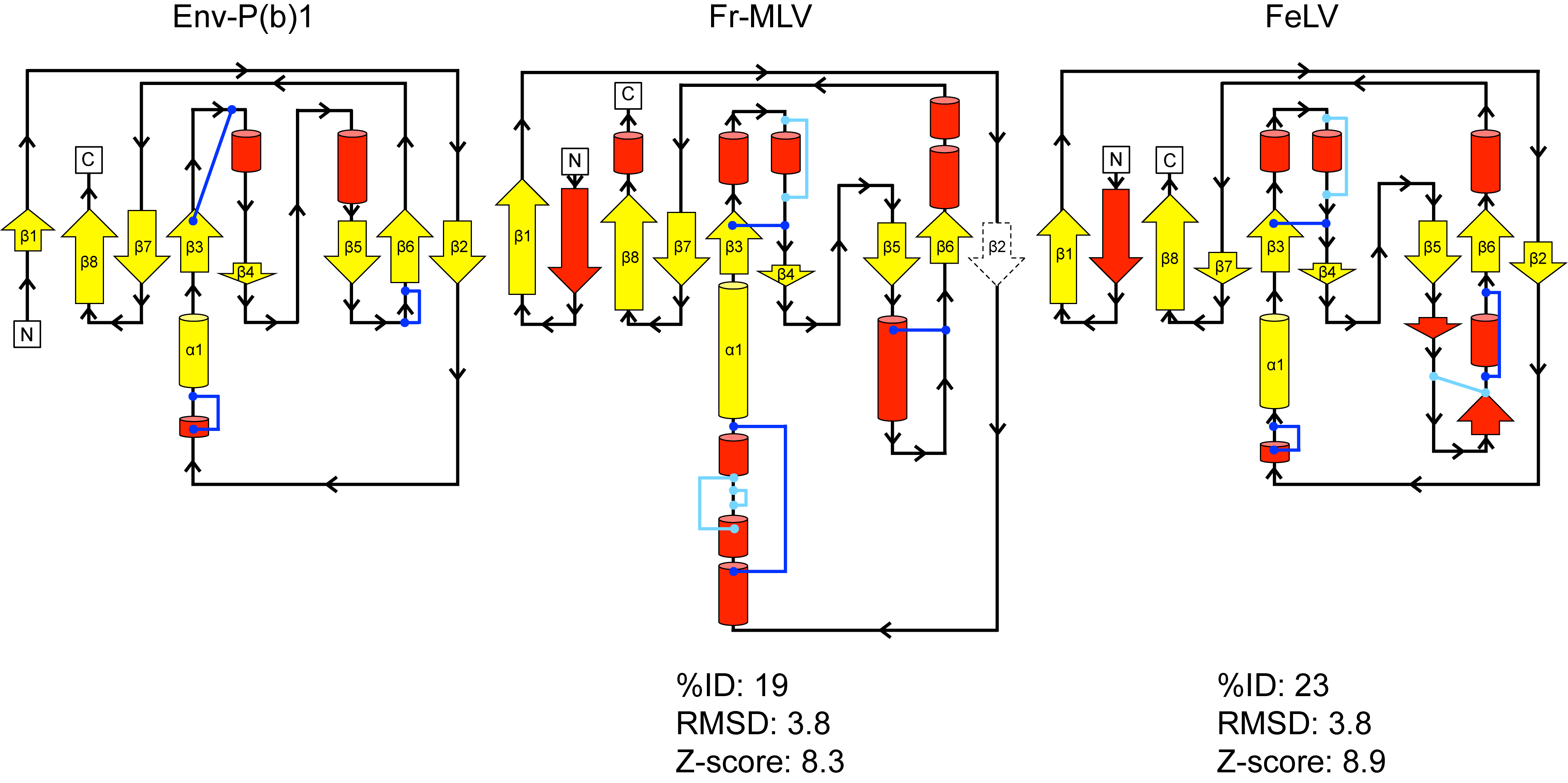

Supplement: FIG S6 [file mBio.02772-20-sf006.tif]
