# Supplementary material for: Structure of the Receptor Binding Domain of EnvP(b)1, an Endogenous Retroviral Envelope Protein Expressed in Human Tissues
Source: mBio. 2020 Nov 17;11(6):e02772-20. doi: 10.1128/mBio.02772-20 (PMC7683403; doi:10.1128/mBio.02772-20)
Supplement: TABLE S2 [file mBio.02772-20-st002.pdf]

Table S2. Sequence information for simian EnvP(b)1 ORFS.

Common name	Latin name	Database	Accession number	Locus	Genome build (if applicable)
African green monkey (Grivet)	*Cercopithecus aethiops*	Genbank	DQ247961	1-1998	
Angola colobus	*Colobus angolensis palliatus*	Ensembl		KN981601:881891-883918	Cang.pa_1.0
Baboon	*Papio Anubis*	UCSC gb		chr7:148757925-148759922	Mar. 2012 (Baylor Panu_2.0/papAnu2)
Black-capped squirrel monkey	*Saimiri boliviensis*	UCSC gb		JH378224:1485877-1487898	Oct. 2011 (Broad/saiBol1)
Black-handed spider monkey	*Ateles geoffroyi*	Genbank	PVHS01003442	16272-18272	
Bolivian titi	*Plecturocebus donacophilus*	Genbank	PVKP010050380	5748-7748	
Bonobo	*Pan paniscus*	UCSC gb		chr14:92597405-92599399	Aug. 2015 (MPI-EVA panpan1.1/panPan2)
Chimpanzee	*Pan troglodytes*	Genbank	DQ247959	1-1998	
Common marmoset	*Callithrix jacchus*	UCSC gb		chr10:118228236-118230215	March 2009 (WUGSC 3.2/calJac3)
Crab-eating macaque	*Macaca fascicularis*	UCSC gb		chr7:157604563-157606560	Jun. 2013 (Macaca_fascicularis_5.0/macFas5)
De Brazzas monkey	*Cercopithecus neglectus*	Genbank	PVKI010048274	9858-11858	
Drill	*Mandrillus leucophaeus*	Ensembl		KN976258:12206340-12208337	Mleu.le_1.0
Emperor tamarin	*Saguinus imperator*	Genbank	PVHO010004519	37081-39063	
Gelada	*Theropithecus gelada*	Ensembl		7b:94045308-94047305	Tgel_1.0
Golden snub-nosed monkey	*Rhinopithecus roxellana*	UCSC gb		KN298779v1:528750-530777	Oct. 2014 (Rrox_v1/rhiRox1)
Gorilla	*Gorilla gorilla gorilla*	UCSC gb		chr14:76616252-76618246	Dec 2014 (gorGor4.1/gorGor4)
Green monkey	*Chlorocebus sabaeus*	Genbank	AQIB01047147.1	159971-61968	
Human	*Homo sapiens*	Genbank	DQ247958	1-1998	
Japanese macaque	*Macaca fuscata fuscata*	Genbank	BFBW01021746	18133-20133	
Mandrill	*Mandrillus sphinx*	Genbank	SRPC01048805	5482077-5484077	
Mantled howler monkey	*Alouatta palliata*	Genbank	PVKV010007614	3795-5807	
Owl monkey	*Aotus nacymaae*	Genbank	XM_012453140	135615 to 137615	
Patas monkey	*Erythrocebus patas*	Genbank	PVJV010049423.1	3447-5444	
Pigtail macaque	*Macaca nemestrina*	Genbank	DQ247962	1-1998	
Proboscis monkey	*Nasalis larvatus*	UCSC gb		chr7:142317833-142319860	Nov. 2014 (Charlie1.0/nasLar1)
Red shanked douc	*Pygathrix nemaeus*	Genbank	PVHW010001312	94580..96595	
Rhesus macaque	*Macaca mulatta*	UCSC gb		chr7:154,780,552-154,782,549	Feb. 2019 (Mmul_10/rheMac10)
Sooty mangaby	*Cercocebus atys*	Ensembl		KQ010752:11226798-11228795	Caty_1.0
Sumatran Orangutan	*Pongo abelii*	UCSC gb		chr14:74295532-74297526	Jan. 2018 (Susie_PABv2/ponAbe3)
Ugandan red colobus	*Piliocolobus tephrosceles*	Genbank	PDMG03000007	13832115-13834145	
White-cheeked gibbon	*Nomascus leucogenys*	Genbank	ADFV01193533.1	11167-13161	
White-faced saki	*Pithecia pithecia*	Genbank	PVIP01006858	91828-93825	
White-fronted capuchin	*Cebus albifroms*	Genbank	PVKJ010010230	27500-29500	
White-headed capuchin	*Cebus imitator*	Ensembl		KV389619:1976940-1978937	Cebus_imitator-1.0
Yellow-cheeked gibbon	*Nomascus gabriellae*	Genbank	DQ247960	1-1995	
