# Supplementary material for: Structure of the Receptor Binding Domain of EnvP(b)1, an Endogenous Retroviral Envelope Protein Expressed in Human Tissues
Source: mBio. 2020 Nov 17;11(6):e02772-20. doi: 10.1128/mBio.02772-20 (PMC7683403; doi:10.1128/mBio.02772-20)
Supplement: TABLE S3 [file mBio.02772-20-st003.pdf]

Table S3. Crystallographic statistics for EnvP(b)1 iRBD

	EnvP(b)1 iRBD	
**PDB ID**	6W5Y	
**Dataset**	Native	Platinum
**Data Collection**	ALS 8.2.2	APS 24-ID-C
Number of datasets	1 Text	1
Resolution, Å	49.56 - 2.5 (2.59 - 2.50)	40.05 - 2.25 (2.33 - 2.25)
Wavelength (Å)	0.9998	1.0719
Space Group	C 1 2 1	C 1 2 1
Unit cell dimensions (a, b, c), Å	243.3 37.4 67.0	243.3 37.4 66.8
Unit cell angles (α, β, γ) °	90 102.3 90	90.00 102.0 90.00
I/σ	12.7 (1.83)	16.0 (2.25)
Rmeas	0.14 (0.93)	0.09 (1.0)
Rpim	0.06 (0.44)	0.034 (0.40)
Rmerge, %	0.13 (0.89)	0.08 (0.91)
CC*	1.0 (0.93)	1.0 (0.96)
CC½	1.0 (0.75)	1.0 (0.87)
Completeness, %	99.9 (99.8)	99.0 (97.5)
Number of reflections	102170 (10047)	185653 (17123)
Redundancy	4.9 (5.0)	6.5 (6.1)
**Phasing**		
Resolution (Å)		3.4
Number of sites		5
FOM (acentric)		0.47
**Refinement**		
Number of reflections:	20893 (2023)	
Working	20881 (2018)	
Free	1011 (90)	
Rwork, %	0.222 (0.321)	
Rfree, %	0.255 (0.346)	
**Ramachandran plot,**		
% (favored, disallowed)	93.7 (0.2)	
Rmsd bond lengths, Å	0.006	
Rmsd bond angles, °	1.17	
Average B-factor	56.2	

Rmerge, Σh,k,lΣi|Ii(hkl) − <I(hkl)>|/Σh,k,lΣiIi(hkl), where I is an intensity that is observed i times; I/σ, signal-to-noise ratio (average observed intensity divided by average standard deviation of the observed intensity); Rwork, Σh,k,l||Fobs| − |Fcalc||/Σh,k,l|Fobs|, where h,k,l cover the “working set” of observed structure factor amplitude (Fobs) reflections used in refinement (total reflections minus the test set) and Fcalc is the calculated structure factor amplitude; Rfree, calculated as for Rwork but on 5% of data excluded before refinement. Values in parentheses refer to highest-resolution shell.
